# Teachers and educators’ experiences and perceptions of artificial-powered interventions for autism groups

**DOI:** 10.1186/s40359-024-01664-2

**Published:** 2024-04-11

**Authors:** Guang Li, Mohammad Amin Zarei, Goudarz Alibakhshi, Akram Labbafi

**Affiliations:** 1 School of History, Capital Normal University, Beijing, China; 2 Allameh Tabataba’i university, Tehran, Iran; 3 Maraghe Branch, PhD Candidate of English Language Teaching, Islamic Azad University, Teheran, Iran

**Keywords:** Artificial intelligence, AI-empowered interventions, Children with autism, Educators, Parents

## Abstract

**Background:**

Artificial intelligence-powered interventions have emerged as promising tools to support autistic individuals. However, more research must examine how teachers and educators perceive and experience these AI systems when implemented.

**Objectives:**

The first objective was to investigate informants’ perceptions and experiences of AI-empowered interventions for children with autism. Mainly, it explores the informants’ perceived benefits and challenges of using AI-empowered interventions and their recommendations for avoiding the perceived challenges.

**Methodology:**

A qualitative phenomenological approach was used. Twenty educators and parents with experience implementing AI interventions for autism were recruited through purposive sampling. Semi-structured and focus group interviews conducted, transcribed verbatim, and analyzed using thematic analysis.

**Findings:**

The analysis identified four major themes: perceived benefits of AI interventions, implementation challenges, needed support, and recommendations for improvement. Benefits included increased engagement and personalized learning. Challenges included technology issues, training needs, and data privacy concerns.

**Conclusions:**

AI-powered interventions show potential to improve autism support, but significant challenges must be addressed to ensure effective implementation from an educator’s perspective. The benefits of personalized learning and student engagement demonstrate the potential value of these technologies. However, with adequate training, technical support, and measures to ensure data privacy, many educators will likely find integrating AI systems into their daily practices easier.

**Implications:**

To realize the full benefits of AI for autism, developers must work closely with educators to understand their needs, optimize implementation, and build trust through transparent privacy policies and procedures. With proper support, AI interventions can transform how autistic individuals are educated by tailoring instruction to each student’s unique profile and needs.

**Supplementary Information:**

The online version contains supplementary material available at 10.1186/s40359-024-01664-2.

## Introduction

Autism education has become an increasingly important area of focus in recent years due to the rising prevalence of autism spectrum conditions (ASC) among children. The estimated prevalence of ASC has increased from 1 in 10,000 in the 1960s to at least 1 in 100 today [1–3]. ASC is a neurodevelopmental condition characterized by impaired social interaction and communication abilities and stereotypical or obsessive behavior patterns. These impairments can significantly impact an individual’s social, educational, and employment experiences, leading to poor long-term outcomes and difficulties in social transactions, independent work, and job fulfillment [4–10].

The reported prevalence of autism spectrum disorders (ASDs) in developed countries is around 2% [11]. ASDs typically manifest within the first three years of life. They are characterized by challenges in social interaction, speech and language delays, avoidance of eye contact, difficulty adapting to changes in the environment, display of repetitive behaviors, and differences in learning profiles [11–13]. Those with ASDs, including children and adults, have a high frequency of anxiety and depression. Neurobiological research has revealed differences in brain development between children with ASDs and neurotypical children [14]. These excessive connections are thought to be due to reduced pruning of damaged neuronal connections during brain development, resulting in disordered neural patterning across the brain and dysregulation in cognitive function coordination between different brain regions [14, 15].

The dominant perspective regarding AI technologies has revolved mainly around understanding these systems as a collection of processes and their corresponding responses, emphasizing autonomy, adaptability, and interactivity [16–21]. These characteristics are considered fundamental technological focuses that researchers argue should be integral to AI systems. Although autonomy, adaptability, and interactivity are significant, they may only cover some essential criteria for an adequate K-12 education. Specifically, these criteria are about skills taught by human educators, such as B. Self-efficacy, technical skills, and socialization skills. Samuel [22] emphasizes that AI technologies should replicate human actions and mimic expressions of “human intelligence, cognition, and logic.” This highlights the need to refine features that determine effective AI in education. The recent challenges in education due to the pandemic provide a unique opportunity to examine the demands on stakeholders, including educators, students, and parents [23–27].

The potential of artificial intelligence (AI) to drive developments in education is well-recognized [6, 7]. Artificial intelligence is one of the technological advancements which can be used in education. AI encompasses a range of technologies that aim to simulate human intelligence, including machine learning, natural language processing, and computer vision [8]. These technologies have already been used in various applications, from speech recognition to image classification, and can potentially revolutionize how we think about education. In the context of autism, AI has the potential to provide personalized learning experiences that are tailored to the specific needs of each child [8]. For example, AI-powered systems can analyze a child’s behavior and responses to stimuli and use this information to adapt the learning materials and activities to suit their needs. Furthermore, AI can also be utilized to support communication and social interaction, which are areas of difficulty for many children with autism [9].

AI-powered interventions in the context of autism education refer to the utilization of artificial intelligence technologies to create tailored and interactive experiences for individuals on the autism spectrum. These interventions encompass a spectrum of applications, including educational tools, therapeutic programs, and support systems designed to address the unique learning and social communication needs of individuals with autism. AI technologies such as machine learning, natural language processing, and computer vision are employed to analyze and respond to the specific behaviors, preferences, and challenges exhibited by each individual [1–6]. The goal is to provide personalized and adaptive learning experiences, enhance social interaction skills, and offer targeted support for cognitive and emotional development. Examples of AI-powered interventions include virtual reality scenarios, interactive games, and educational software that can dynamically adjust content based on real-time feedback, creating a more individualized and effective educational approach for children with autism [2–5].

Moreover, there is a risk of bias and discrimination in AI-powered interventions for children with autism. For example, if the AI system is trained on data that is not representative of the diverse population of children with autism, it may not be effective for all individuals [10]. Moreover, there is a risk of perpetuating harmful stereotypes or reinforcing inappropriate behaviors if the AI system is not designed and programmed with ethical considerations (10). Third, there are concerns about data privacy and security when using AI in education for children with autism. For instance, if sensitive personal information is collected and stored by the AI system, there is a risk that it could be misused or accessed by unauthorized parties [16]. Therefore, it is essential to address these challenges and concerns to fully realize the potential of AI in education for children with autism. By doing so, we can create evidence-based and ethically sound interventions that support personalized learning and social communication skills while mitigating the risks associated with AI-powered education.

The potential of AI in autism education lies in its ability to offer personalized learning experiences, tailoring interventions to the unique needs of each child [8]. By analyzing a child’s behavior and responses, AI can adapt learning materials, potentially revolutionizing education for children with autism. However, this transformative potential is not without challenges. The risk of bias and discrimination looms large, as AI systems may not be effective if trained on non-representative data, perpetuating harmful stereotypes [10]. Ethical considerations become paramount, addressing concerns about data privacy and security, which, if overlooked, pose potential risks associated with unauthorized access and misuse of sensitive information [16]. Bridging the gap between the promise of AI in education and its responsible application is crucial. Therefore, this study aims to explore educators’ experiences and perceptions of AI-powered interventions for autism, shedding light on the nuanced landscape where technological advancements intersect with the delicate realm of autism education.

### Research questions

In line with the research gap mentioned in the previous section, the following research questions are raised:

1. What are the benefits and challenges of using AI-powered interventions to support the learning and social communication skills of children with autism from teachers’ and educators’ perceptions?
2. How can AI-powered interventions be designed and implemented to ensure that they are culturally and linguistically appropriate for a diverse population of children with autism while also avoiding bias and discrimination in the learning materials and activities?

## Review of literature

### Theoretical background

Machine learning is a component of artificial intelligence (AI) wherein models perform tasks autonomously without human intervention. Traditional machine learning models are trained using input data, enabling accurate outcome predictions. Deep learning, a subset of machine learning, employs extensive data to prepare models, achieving similarly high prediction accuracies. Both models are frequently utilized in diagnosing neurological disorders such as autism [28, 29], ADHD [30, 31], and depression [32, 33]. Diagnostic inputs encompass images from computerized tomography (CT), magnetic resonance imaging (MRI), positron emission tomography (PET) scans, or electroencephalogram (EEG) signals.

AI has been instrumental in social skills training for children with autism spectrum disorders (ASDs), aiding in recognizing and responding to social cues. Belpaeme et al. [34] utilized sensory features (facial expressions, body movements, and voice recordings) as inputs to a machine-learning model implemented in a robot for analyzing autistic children’s behavior and engagement levels during therapy. This study demonstrated the robot’s potential to adapt to interactants, influencing engagement. Another survey by Sanghvi et al. [35] employed postural expressions, specifically silhouette images of the upper body during chess playing, to analyze the engagement levels of autistic children. The integration of representative data with an affect recognition model suggested the potential for the robot to serve as a game-mate for autistic children in real-world scenarios. Kim et al. [36] employed audio recordings to assess the emotional states of autistic children, enhancing the robot’s ability to evaluate engagement and modify responses for a more interactive learning environment.

Various studies explored diverse input features such as facial expressions [37], body movements [38], and biosignals [39]. Esteban et al. [40] investigated facial expressions, direction of look, body posture, and voice tones as input features to a model within the NAO robot for assessing the social engagement of autistic children, showcasing the capability of robots to possess increased autonomy. Rudovic et al. [41] developed a personalized deep model using coordinated video recordings, audio recordings, and biosignals to assess engagement in autistic children, outperforming non-personalized machine learning solutions. Another study created a hybrid physical education teaching tool using speech recognition and artificial intelligence, achieving a recognition accuracy of over 90% for a voice interactive educational robot. Collectively, these studies affirm that AI holds promise in enhancing social interaction and supportive education for children with mental disorders.

### Artificial intelligence and education

The use of AI technology in education has led to increased published studies on the subject, with a reported growing interest and impact of research on AI in education [42]. AI literacy, which refers to the capacity to comprehend the essential processes and concepts underpinning AI in various products and services, has been discussed in several studies [43–47]. Ng et al. [48] proposed a four-dimensional AI literacy framework covering knowing and understanding AI, using and applying AI, evaluating and creating AI, and AI ethics.

Recent review papers on AI in education have highlighted several major AI applications, such as intelligent tutoring systems, natural language processing, educational robots, educational data mining, discourse analysis, neural networks, affective computing, and recommender systems [22, 23, 33–34]. However, Chen et al. [49] identified some critical issues in their review paper on AI in education, including a lack of effort in integrating deep learning technologies into educational settings, insufficient use of advanced techniques, and a scarcity of studies that simultaneously employed AI technologies and delved extensively into educational theories. Furthermore, there needs to be more knowledge and discussion on the role of AI in early childhood education (ECE), an area often ignored in cutting-edge research.

### Using AI to teach children with ASD

Autism Spectrum Disorder (ASD) is a complex neurodevelopmental disorder that affects communication, social interaction, and behavior (1). The disorder is characterized by various symptoms and severity levels, making it challenging to provide effective interventions for affected individuals [12]. Children with ASD often experience difficulties in learning and require specialized educational interventions to help them achieve their full potential [1]. In recent years, there has been growing interest in the potential of AI to improve the learning outcomes of children with autism [8). AI has the potential to provide personalized learning experiences that are tailored to the specific needs of each child with autism [9]. For example, AI-powered systems can analyze a child’s behavior and responses to stimuli and use this information to adapt the learning materials and activities to suit their needs [8].

AI can also be used to support communication and social interaction, which are areas of difficulty for many children with autism [10]. Chatbots and virtual assistants can provide a non-judgmental and non-threatening environment for children to practice their social skills while providing feedback and guidance [23]. These interventions can be particularly valuable for children who struggle with face-to-face interactions or feel uncomfortable in social situations [24]. Despite the potential benefits of using AI in education for children with autism, several challenges and concerns need to be addressed:

First, there is a lack of consensus on the most effective ways to use AI to support learning for autistic children [8]. While there have been some promising results from initial studies, more research is needed to determine the most effective methods for using AI to personalize learning and support social communication skills in this population [10]. Second, there is a risk of bias and discrimination in AI-powered interventions for children with autism. For example, if the AI system is trained on data that is not representative of the diverse population of children with autism, it may not be effective for all individuals [9]. Moreover, there is a risk of perpetuating harmful stereotypes or reinforcing inappropriate behaviors if the AI system is not designed and programmed with ethical considerations [23]. And, third, there are concerns about data privacy and security when using AI in education for children with autism. For instance, if sensitive personal information is collected and stored by the AI system, there is a risk that it could be misused or accessed by unauthorized parties [10].

Several research studies have investigated the use of AI in education for children with autism. For example, Goodwin and Stone [8] developed an AI-powered system called Maki, which uses natural language processing to provide personalized feedback on social communication skills. The system was effective in improving social communication skills in children with autism. Similarly, Alzoubi et al. [50] developed an AI-powered system that uses virtual reality to provide social skills training for children with autism. The system was found to be effective in improving social skills and reducing anxiety in children with autism.

Other research studies have explored the potential of AI to improve different aspects of learning for children with autism. For example, Zhang et al. [10] developed an AI-assisted system that uses computer vision and machine learning to provide personalized feedback on handwriting skills. The system was effective in improving handwriting skills in children with autism. Similarly, Wang et al. [51] developed an AI-powered system that uses game-based learning to enhance math skills in children with autism.

There have also been efforts to develop AI-powered systems that can assist teachers and parents in providing effective interventions for children with autism. The system effectively improved the quality of interventions offered by teachers and parents. However, there are also concerns about the potential negative impacts of AI on children with autism. For example, some studies have suggested that excessive use of AI-powered interventions could reduce face-to-face interactions and social skills development [9]. Additionally, there are concerns about the potential for AI-powered interventions to replace human teachers and therapists, which could have negative implications for the quality of care provided to children with autism [8].

To address these concerns and maximize the potential benefits of AI for children with autism, it is essential to prioritize ethical considerations and involve stakeholders in designing and implementing AI-powered interventions [23]. This includes ensuring that AI systems are developed and programmed to avoid bias and discrimination, protecting the privacy and security of personal data, and promoting transparency and accountability in using AI in education for children with autism [10].

Other studies have investigated using chatbots and virtual assistants to support social communication skills in children with autism. For example, Kocaballi et al. [52] developed a chatbot called Tess that provides social skills training and support for children with autism. The chatbot was effective in improving social communication skills in children with autism. Similarly, Tanaka et al. [53] developed a virtual assistant called Miko that uses artificial empathy to support social communication skills in children with autism.

Further studies highlighted the importance of ethical consideration while using AL in education for children with autism. For example, there is a risk of perpetuating harmful stereotypes or reinforcing inappropriate behaviors if the AI system is not designed and programmed with ethical considerations [23]. Moreover, there is a risk of bias and discrimination if the AI system is trained on data that is not representative of the diverse population of children with autism [9]. Therefore, it is essential to carefully consider the ethical implications of using AI in education for children with autism. In conclusion, utilizing AI in education can transform how we think about learning and support children with autism to achieve their full potential.

## Research Methodology

### Informants

The study used purposive sampling to select 20 informants who met specific criteria. These individuals were parents or educators of autistic children and had valuable experience using AI-powered interventions to improve their children’s learning and social communication skills. They were all Iranian living in Tehran, Iran. 30% (*n* = 6) were female and 70% (*n* = 14) were male.

The participants in the study encompassed an age range spanning from 29 to 58 years old. Educators teaching experience was above 8 years. Recruitment efforts were conducted through various channels and social media platforms to ensure a diverse and representative sample. Potential participants were fully informed about the study’s purpose, procedures, and possible benefits throughout the recruitment process. They were also told of their rights as participants and the assurance of confidentiality. To confirm their willingness to participate, informants were asked for written consent before formal inclusion in the study.

### Data collection

The study used semi-structured interviews and focus groups to collect data from the informants. The researcher developed the interview questions (Appendix), and a panel of three qualitative researchers reviewed their relevance. Interviews were conducted individually, either in person or virtually, and lasted approximately 45–60 min each. Focus groups with 3–5 participants conducted almost or in person were also organized. The duration of the focus group discussions was between 60 and 90 min. During the data collection process, the interviews and focus group sessions were audio-recorded to capture participants’ responses and insights accurately. These recordings were later transcribed verbatim, allowing a comprehensive analysis of the data collected. Through semi-structured interviews and focus groups, the study aimed to obtain complete and detailed information about participants’ experiences and perspectives regarding using AI-assisted interventions to support the learning and social communication skills of children with autism. The semi-structured nature of the interviews allowed for flexibility in exploring different topics while ensuring a consistent data collection framework for all participants. Additionally, the dynamic and interactive nature of the focus groups encouraged group discussions and allowed participants to share and build on one another.

### Data analysis

Following the data collection phase, the study thoroughly analyzed the information collected. The audio recordings of the interviews and focus group sessions were transcribed verbatim, resulting in a comprehensive text dataset that captured participants’ responses and insights. The analysis began with a thorough familiarization process in which researchers immersed themselves in the transcribed data to understand participants’ accounts deeply. This immersion allowed researchers to identify recurring themes, patterns, and noteworthy information in the data set. A systematic analysis approach was used to ensure reliability and validity. Data were coded using a combination of inductive and deductive methods. First, an open coding process was conducted in which researchers generated initial codes by closely examining the data and labeling meaningful segments. As the analysis progressed, these codes were refined, grouped, and organized into categories and subcategories, creating a coding framework. After coding, researchers conducted a thematic analysis by identifying overarching themes from the data. The topics represented vital concepts, ideas, and perspectives shared by participants regarding the use of AI-assisted interventions to support the learning and social communication skills of children with autism. Throughout the analysis, the researchers ensured the accuracy and trustworthiness of the findings by employing techniques such as member checking, where participants were allowed to review and validate the interpretations made from their data.

### Ethical considerations

The study adhered to ethical guidelines for conducting research with human subjects. Informed consent was obtained from all participants. Participants’ privacy and confidentiality were protected throughout the research process. The study also obtained ethical clearance from a relevant research ethics committee.

### Findings

The study’s findings were presented in a report summarizing the themes and sub-themes that emerged from the data analysis. The report also provides recommendations for designing and implementing culturally and linguistically appropriate AI-powered interventions for children with autism while avoiding bias and discrimination in the learning materials and activities. The report also includes direct participant quotes to illustrate their experiences and perceptions. The findings are presented based on the order of research questions,

#### Benefits and challenges of AI-powered interventions

Informants of the study mentioned three benefits and some challenges of AI-empowered intervention for children with autism. Each is explained and exemplified as follows.

##### Increased engagement and motivation among children with autism

AI-powered interventions can use technologies like robots, virtual reality, and interactive games to provide personalized and engaging experiences for children with autism. Informants believed that AI-powered interventions can effectively increase engagement and motivation among children with autism. For example, educator 1 stated, “Children with autism who interacted with a humanoid robot showed increased engagement and motivation compared to those who received traditional therapy.” Educator 5 said, “By leveraging AI technologies, interventions for children with autism can be tailored to their needs and preferences, providing a more personalized and engaging learning experience. This can lead to improved outcomes and better quality of life for children with autism and their families. This finding is also supported by parent one, who stated, “My son used to struggle with traditional teaching methods, but with AI-powered interventions, he is more engaged and motivated to learn. The technology provides him with immediate feedback, which helps him understand his mistakes and learn from them.”

##### Customized and individualized interventions that cater to the unique needs of each child

Informants argued that every child with autism is unique, with their own set of strengths and challenges. Therefore, interventions tailored to each child’s specific needs and preferences can be more effective in promoting their development and well-being. This finding echoes the direct quotation by educator 6 who stated, “One size does not fit all when it comes to autism interventions. Each child is unique and requires a personalized approach that takes into account their individual strengths, challenges, and interests.” (Educator 6). Similarly, parent 6 stated, “As a parent, I have learned that the key to helping my child with autism is to focus on his individual needs. By working with his teachers and therapists to develop a personalized intervention plan, we have seen significant progress in his development and well-being.”

##### Real-time feedback to both children and educators about progress and areas for improvement

Real-time feedback involves providing immediate and ongoing information about a child’s performance and progress in a given activity or intervention. This feedback can reinforce positive behaviors, correct errors, and identify areas where additional support or instruction may be needed. Real-time feedback can be especially beneficial for children with autism, who may benefit from more frequent and targeted feedback to support their learning and development. By providing timely and specific feedback, children with autism can better understand their strengths and areas for improvement, and educators can adjust their interventions and supports accordingly. As an example, one of the educators stated, “Real-time feedback is crucial in helping children with autism learn and grow. By providing immediate and targeted feedback, we can reinforce positive behaviors and help children build new skills.” (Educator 4). Another educator stated, “Real-time feedback is not just important for children but for educators as well. By receiving ongoing feedback about a child’s progress, we can make more informed decisions about the interventions and supports that are most effective for them.“(Educator 8).

##### The potential for AI-powered interventions to enhance the work of educators and provide them with additional tools and resources

AI-powered interventions have the potential to enhance the work of educators and provide them with additional tools and resources to support the learning and development of children with autism. AI technologies like machine learning algorithms and natural language processing can analyze and interpret data from various sources, including assessment results, behavioral observations, and social communication interactions. This can provide educators with valuable insights and information about each child’s strengths, challenges, and learning needs. Educator 10 stated, “AI-powered interventions can provide educators with powerful tools and resources for supporting autistic children. By analyzing data and providing real-time feedback, these interventions can help educators tailor their teaching strategies and supports to the unique needs of each child.” Educator 3 also stated,” AI-powered interventions have the potential to transform the way we support children with autism in the classroom. By providing educators with insights and information about each child’s learning needs, these interventions can help us deliver more effective and personalized instruction.”

#### Challenges of AI-powered interventions

The content of interviews with informants was analyzed, and five main themes were extracted. Each is explained and exemplified as follows.

##### Lack of personalization

Informants stated that while AI-powered interventions have the potential to be personalized, there is a risk that they may not account for the unique needs and preferences of each child. For example, educator 3 stated, “We need to remember that technology is a tool, not a replacement for human interaction.”

##### Limited access to technology

Not all families and schools can access the necessary technologies for AI-powered interventions. As a parent of a child with autism notes, “Technology can be expensive, and not all families can afford it.”

##### Difficulty in interpreting and responding to social cues

Children with autism may have trouble analyzing and reacting to social cues, making it challenging to interact with AI technologies. A clinical psychologist notes: “Children with autism may struggle to understand that a robot or virtual character is not a real person, which can limit the effectiveness of AI-powered interventions.”

##### Ethical concerns

Ethical concerns surrounding using AI technologies with children include privacy, data security, and the potential for misuse or unintended consequences. The Director of Education at one School for Children with Autism notes: “We need to be mindful of the potential risks and unintended consequences of using AI technologies with children with autism.”

##### Lack of human interaction

While AI-powered interventions can be engaging and interactive, they cannot replace the importance of human interaction in promoting social and emotional development in children with autism. As a parent of a child with autism notes: “Technology can be helpful, but it is important to balance it with real-life experiences and interactions.”

##### Concerns about the cost and affordability of these interventions

One concern related to using interventions for children with autism is their cost and affordability. Many interventions, such as behavioral and developmental therapies, assistive technologies, and specialized education programs, can be expensive and may not be covered by insurance or other funding sources. This can create barriers for families, particularly those with limited financial resources, in accessing the interventions their child needs to thrive. As Educator 9 stated, “The cost of interventions for children with autism can be a significant burden for families, particularly those with limited financial resources. We must ensure these interventions are accessible and affordable for all families.” Similarly, parent 5 stated, “As a parent of a child with autism, the cost of interventions has been a major concern for our family. Based on our financial limitations, we have had to decide which interventions to prioritize.”

#### Suggestions for improving the quality of AL-empowered interventions

Interviews with informants were thematically analyzed, and different themes were extracted. Each theme is explained and exemplified as follows.

##### Using culturally and linguistically appropriate interventions

Participants emphasized the importance of designing and implementing AI-powered interventions that are culturally and linguistically appropriate for a diverse population of children with autism. Some of the suggestions made by participants include:

- Ensuring that the language and content of the interventions are culturally sensitive and relevant to the target population.
- Incorporating diverse perspectives and experiences into the design and development process.
- Providing interventions in multiple languages to accommodate diverse linguistic backgrounds.

Quotations from educators and parents support these suggestions. For instance, educator 1 stated, “Cultural sensitivity is important when designing interventions for children with autism, particularly for those from diverse backgrounds. We need to ensure that the interventions are culturally relevant and take into account the unique needs and experiences of each child.” Similarly, parent 6 stated, “As a parent of a child with autism who comes from a different cultural background, I appreciate interventions that take into account my child’s unique needs and experiences. It’s important to have interventions that are culturally sensitive and relevant.”

##### Avoiding bias and discrimination

Participants also emphasized the importance of avoiding bias and discrimination in AI-powered interventions’ learning materials and activities. Some of the suggestions made by participants include:

- Conducting regular audits of the interventions to identify and address any potential biases or discriminatory content.
- Incorporating diverse perspectives and experiences into the design and development process to avoid perpetuating stereotypes.
- Providing training and education to educators and developers to ensure that they are aware of and can address potential biases and discrimination.

Quotations from informants support these strategies. As an example, educator 8 stated,

“We need to be careful to avoid stereotypes and biases in the interventions we design and implement. It’s important to be aware of potential biases and to work to address them.” Similarly, parent 7 stated, “To ensure that AI-powered interventions are effective and inclusive, we need to make sure that they are designed with diversity and inclusivity in mind. This means avoiding discrimination and bias in the materials and activities.”

##### Training educators

Participants discussed the role of educators in implementing AI-powered interventions to support the learning and social communication skills of children with autism. Some of the key findings include:

- The importance of providing training and education to educators to ensure that they can effectively implement these interventions.
- The need for educators to work collaboratively with parents and other professionals to ensure that the interventions are tailored to the unique needs of each child.

“Educators play a critical role in implementing AI-powered interventions. They need to be trained and educated on how to use these interventions effectively and how to tailor them to the unique needs of each child.” [Educator 3).

##### We regularly audit the interventions to identify and address potential biases or discriminatory content

Conducting regular audits of interventions for children with autism is an essential step in ensuring that these interventions are effective, evidence-based, and free from biases or discriminatory content. Regular audits help identify areas for improvement, ensure that interventions are aligned with current best practices and ethical guidelines, and promote accountability and transparency in developing and implementing these interventions. Here are two quotations that address the importance of conducting regular audits of interventions for children with autism. To exemplify this finding, the following quotations are presented:

“As educators and researchers, it is our responsibility to ensure that interventions for children with autism are evidence-based, effective, and free from biases or discriminatory content. Regular audits can help us identify and address any areas of concern and promote the highest standards of quality and ethical practice.” (Educator 4).

“Regular audits are essential to ensuring that interventions for children with autism are meeting the needs of all children, regardless of their race, ethnicity, gender, or other factors. We must be vigilant in identifying and addressing any biases or discriminatory content that may be present, and work to create interventions that are inclusive and equitable for all children.” (Educator 9).

##### Involving families and communities in the design and implementation process ensures their voices and perspectives are heard and valued

Involving families and communities in the design and implementation process of interventions for children with autism is crucial to ensuring that their voices and perspectives are heard and valued. Families and communities can provide valuable insights and feedback on the needs and preferences of children with autism and the effectiveness and cultural relevance of interventions. Here are two quotations that address the importance of involving families and communities in the design and implementation process:

“Families and communities are essential partners in the design and implementation of interventions for children with autism. Their insights and feedback can help us create interventions that are effective, culturally relevant, and responsive to the needs of all children.” (Educator 10).

“As a parent of a child with autism, I know firsthand the importance of involving families and communities in the design and implementation of interventions. By listening to our voices and perspectives, researchers and educators can create interventions that are more meaningful and effective for our children.” (Parent 8).

## Discussion and implications

The present study aimed at exploring the teachers and educators’ experiences and perceptions of artificial intelligence powered interventions for Autism groups. A qualitative research study was employed and interviews were analyzed thematically and different themes were extracted. Participant believed that AI-powered interventions represent a groundbreaking frontier in reshaping the support systems for the learning and social communication skills of children with autism [54]. Participants also highlighted several noteworthy benefits, with a critical emphasis on the heightened engagement and motivation witnessed among children with autism when exposed to AI-powered interventions [1, 2, 54]. Recognizing the limitations of traditional teaching methods in meeting the distinctive learning needs of these children, AI interventions emerge as a promising avenue [1, 2].

The first advantage underscored by participants is the adaptability of AI-powered interventions to provide personalized and individualized support, furnishing real-time feedback to children and educators regarding progress and areas for improvement [3–5]. This tailored approach aligns seamlessly with the diverse and unique challenges presented by children with autism. However, embracing AI-powered interventions is full of challenges, and participants articulated various concerns [55, 56]. Technical glitches and difficulties were identified as potential disruptors of the learning process, prompting apprehensions about an overreliance on technology [55, 56]. Moreover, the limited access to technology and resources in specific communities and regions raises concerns about the equitable distribution of intervention benefits [55, 56]. Addressing these challenges is imperative to ensure that all children with autism, irrespective of geographical location or socioeconomic status, have equitable access to effective interventions.

The second theme, cultural and linguistic appropriateness, emerged as a primary consideration, with participants highlighting the importance of interventions tailored to the diverse backgrounds of children with autism [55, 56]. This includes ensuring that the language and content of interventions are culturally sensitive and relevant, integrating diverse perspectives into the design process, and providing interventions in multiple languages ​​to accommodate linguistic diversity [7–9]. This finding is consistent with the findings of the previous research which highlighted that language differences can pose significant barriers to accessing autism interventions, highlighting the urgent need for interventions in the child’s native language [66].

As the third extracted theme “mitigating bias and discrimination in AI-powered interventions” extracted as another critical aspect, necessitating regular audits to identify and rectify potential biases [57]. The imperative of incorporating diverse perspectives into the design process and providing training to educators and developers to address biases and discrimination was highlighted as crucial [10, 11]. This finding confirms the findings of the study that emphasizes the pivotal role of involving families and communities in designing and developing autism interventions to ensure cultural sensitivity and effectiveness [67].

Despite the above-mentioned potential of AI-powered interventions, the participants concurrently acknowledged the need for further research to evaluate the effectiveness of remote interventions and ensure their cultural and linguistic appropriateness [12, 13]. Simultaneously, there are apprehensions and concerns with the potential for these interventions to exacerbate existing disparities in access to care if not implemented equitably. Moreover, challenges have been discerned alongside these benefits, prompting a comprehensive approach to ensure effectiveness, inclusivity, and accessibility [55, 56]. Technical glitches, concerns about overreliance on technology, and limited access to resources pose hurdles that need addressing [55, 56]. Policymakers must prioritize equitable access, focusing on both technological infrastructure and training programs for educators [55, 56].

In addition, ensuring cultural and linguistic appropriateness emerges as a critical consideration in designing and implementing AI-powered interventions [55, 56]. Culturally sensitive content, diverse perspectives in development, and multilingual offerings are underscored as essential [7–9]. Recognizing potential biases and discrimination, participants advocate for regular audits, diversity in development teams, and education on bias mitigation as integral components of ethical AI intervention deployment [10, 11, 57].

## Conclusions

AI-powered interventions have emerged as a promising avenue to revolutionize the support for children with autism, offering transformative benefits while presenting challenges that demand careful consideration [54]. One pivotal advantage emphasized by participants is the heightened engagement and motivation observed among children with autism undergoing AI-powered interventions [54]. This is particularly noteworthy as traditional teaching methods often need to catch up in meeting the unique learning needs of these children. AI interventions, utilizing technologies such as robots, virtual reality, and interactive games, create personalized and engaging experiences, as reported by educators and parents.

It can also be concluded that transformative potential of AI-powered interventions underscores the need for collaborative efforts among educators, parents, and developers, ensuring effectiveness, inclusivity, and accessibility for all children [60–65]. The imperative of providing interventions in multiple languages and incorporating diverse perspectives into the design and development process is underscored [63]. Additionally, including culturally responsive teaching practices alongside AI interventions emerges as a strategy to enhance engagement and outcomes, particularly for children from diverse cultural backgrounds [68]. Ongoing research, collaborative endeavors, and an unwavering commitment to addressing challenges are imperative to maximize the benefits of AI-powered interventions for children with autism.

It can also be inferred that the collaborative involvement of families and communities is championed to enhance interventions’ impact and cultural sensitivity [12, 13, 67]. Balancing technology with human interaction is deemed crucial, emphasizing the irreplaceable role of personal connections in social and emotional development [39, 41]. Moreover, the potential for AI-powered interventions to address access disparities, especially in remote or underserved areas, highlights the importance of further research and evaluation [58, 59]. However, concerns persist about exacerbating existing disparities, demanding meticulous attention to cultural, linguistic, and regional nuances.

As another concluding remark, it can be inferred that AI-powered interventions have the potential to revolutionize the way we support the learning and social communication skills of children with autism. These interventions can provide customized and individualized interventions that cater to the unique needs of each child, providing real-time feedback to both children and educators about progress and areas for improvement. AI-powered interventions can also improve access to care for children with autism, particularly for those in remote or underserved areas. The findings suggest that to ensure that AI-powered interventions are culturally and linguistically appropriate for a diverse population of children with autism while also avoiding bias and discrimination in the learning materials and activities, it is essential to incorporate various perspectives and experiences into the design and development process, provide interventions in multiple languages, ensure that the language and content of the interventions are culturally sensitive and relevant, deliver training and education to educators and developers, conduct regular audits of the interventions, involve families and community members in the design and implementation process, and use culturally responsive teaching practices. These efforts can help to address the challenges and considerations of using AI-powered interventions and ensure that all children with autism have access to practical, inclusive, and culturally appropriate interventions.

However, several challenges and considerations need to be taken into account to ensure that these interventions are effective, inclusive, and accessible to all children with autism. These challenges include technical difficulties, overreliance on technology, limited access to technology and resources in specific communities and regions, and the need to design and implement culturally and linguistically appropriate interventions to avoid bias and discrimination.

Finally, one recurring theme is the importance of professional development for educators, which recognizes their critical role in successfully applying AI-powered interventions. Providing educators with technological expertise, cultural sensitivity, and ethical awareness is essential. Furthermore, legislators, educators, and parents must work together to prioritize the financial accessibility of interventions. The ramifications in this complex environment suggest a comprehensive and collaborative strategy. The key to success is overcoming obstacles, adopting technology responsibly, and giving accessibility and inclusivity top priority in intervention and education initiatives. Because technology constantly changes, we must remain committed to ongoing iteration and improvement. Community, parent, and educator feedback loops help us refine AI-powered interventions.

### Limitations and suggestions for further studies

The current body of research on AI-powered interventions for children with autism, while promising, grapples with several limitations that warrant careful consideration. Firstly, the generalization of findings remains a challenge, as many studies tend to focus on specific demographic groups or particular manifestations of autism spectrum disorder (ASD). This limits the broader applicability of the insights gained, as the diversity within the autism spectrum may not be comprehensively represented. Additionally, a notable gap exists in understanding the long-term efficacy of AI interventions. While short-term outcomes are frequently explored, there is a scarcity of research delving into the sustained impact of these interventions on the developmental trajectories of children with autism. Longitudinal studies are crucial to elucidating AI-powered approaches’ durability and lasting benefits.

Moreover, the current literature may lack ethnic and cultural diversity, raising concerns about AI interventions’ universal applicability and artistic sensitivity. This underrepresentation hinders our understanding of how these technologies might function across diverse populations. Ethical considerations, although acknowledged, need to be thoroughly examined. Privacy, data security, and potential biases in algorithmic decision-making demand a more in-depth investigation to ensure responsible and equitable use of AI technologies in educational settings.

To address these limitations, future research should prioritize several vital areas. Long-term impact assessments are imperative to ascertain the sustained efficacy of AI interventions over time. Diverse and inclusive studies encompassing a range of ethnicities and cultural backgrounds are essential to validate the universal applicability of these technologies. Robust ethical frameworks should be developed to guide the implementation of AI interventions, addressing privacy, security, and bias concerns. Comparative studies, pitting AI interventions against traditional methods, will offer nuanced insights into their relative advantages and limitations. Family and community involvement in designing and implementing AI interventions should be explored further, recognizing the unique insights these stakeholders bring. Finally, comprehensive cost-benefit analyses are necessary to evaluate the economic aspects of AI interventions, ensuring their affordability and long-term viability in diverse educational settings. In navigating these avenues, researchers can contribute substantively to the responsible and inclusive integration of AI-powered interventions for children with autism.

### Electronic supplementary material

Below is the link to the electronic supplementary material.

Supplementary Material 1

## Data Availability

The data will be made available upon request from the corresponding author (Corresponding author: email: alibakhshi@atu.ac.ir.
