# Supplementary material for: Teachers and educators’ experiences and perceptions of artificial-powered interventions for autism groups
Source: BMC Psychol. 2024 Apr 11;12:199. doi: 10.1186/s40359-024-01664-2 (PMC11010416; doi:10.1186/s40359-024-01664-2)
Supplement: Supplementary file 1 — Supplementary Material 1 [file 40359_2024_1664_MOESM1_ESM.docx]

Interview Checklist

1. Can you please describe your background and experience in working with students or individuals with autism spectrum disorder (ASD)?
2. What motivated you to explore or use Artificial Intelligence-powered interventions in your work with Autism groups?
3. Could you elaborate on the specific Artificial Intelligence tools or technologies you have integrated into your teaching or support practices for individuals with ASD?
4. How do you perceive the effectiveness of these Artificial Intelligence interventions in addressing the unique needs and challenges of Autism groups? Could you share any success stories or outcomes you've observed?
5. In your opinion, what are the advantages and limitations of utilizing Artificial Intelligence technologies in Autism interventions compared to traditional approaches?
6. How do you adapt or customize the Artificial Intelligence interventions to cater to the individual needs and preferences of students with ASD?
7. What kind of training or professional development have you received to effectively implement these interventions? How do you stay updated on the latest developments in this field?
8. Have you encountered any challenges or obstacles while using Artificial Intelligence-powered interventions with Autism groups? How did you address or overcome these challenges?
9. Can you share your views on the ethical considerations related to using technology, particularly Artificial Intelligence, in Autism interventions? How do you ensure that the interventions respect the individual's dignity and autonomy?
10. In your interactions with students or individuals with ASD, have you noticed any changes in engagement, communication, or learning outcomes due to the integration of Artificial Intelligence technologies?
11. How do you collaborate with other educators, professionals, and caregivers when implementing these interventions? What role does interdisciplinary teamwork play in enhancing the effectiveness of these interventions?
12. Looking ahead, how do you envision the future of Artificial Intelligence-powered interventions for Autism groups? What improvements or advancements would you like to see in this field?
